# 鹿龙再生汤通过调控Jak2/Stat3/Acsl4途径改善再生障碍性贫血小鼠的造血功能

**DOI:** 10.3760/cma.j.cn121090-20241129-00493

**Published:** 2025-06

**Authors:** 诚程 周, 晓鋆 吴, 玮 孙, 甜滋 盛, 红 刘

**Affiliations:** 1 南通大学医学院，南通 226000 Medical School, Nantong University, Nantong 226000, China; 2 南通大学附属医院，南通 226000 Affiliated Hospital of Nantong University, Nantong 226000, China; 3 常熟市中医院，常熟 215500 Changshu Hospital of Traditional Chinese Medicine, Changshu 215500, China

**Keywords:** 再生障碍性贫血, 鹿龙再生汤, Jak2/Stat3信号通路, Acsl4, 骨髓造血功能, Aplastic anemia, Lulongzaisheng decoction, Jak2/Stat3 signaling pathway, Acsl4, Bone marrow hematopoietic function

## Abstract

**目的:**

探讨鹿龙再生汤对再生障碍性贫血（AA）模型小鼠铁死亡的影响及其对AA的潜在作用机制。

**方法:**

以近交系雌性BALB/c小鼠作为对照（对照组），应用IFN-γ腹腔注射联合白消安灌胃的方法建立AA小鼠模型组，并使用鹿龙再生汤对AA组连续灌胃10 d治疗，评估其对Jak2/Stat3/Acsl4信号通路的影响。胸骨组织病理学观察各组小鼠骨髓损伤程度；流式细胞术检测骨髓细胞中脂质活性氧（ROS）的产生；免疫组织化学染色观察胸骨中4-HNE的表达；Western blot法分析Jak2、p-Jak2、Stat3、p-Stat3及Acsl4的蛋白表达水平；ELISA法检测外周血IL-6水平。

**结果:**

与对照组相比，AA组小鼠骨髓腔内造血组织减少，脂肪化明显；AA+鹿龙再生汤组小鼠骨髓脂肪化减少。对照组、AA组、AA+鹿龙再生汤组骨髓细胞脂质ROS比例分别为（47.01±3.07）％、（53.81±1.99）％、（49.50±3.98）％（*P*<0.05），AA+鹿龙再生汤组的脂质ROS累积量较AA组略低。对照组、AA组、AA+鹿龙再生汤组骨髓腔内4-HNE的表达面积分别为（6.34±1.07）％、（35.26±3.68）％、（16.97±1.30）％（*P*<0.05），与AA组相比，AA+鹿龙再生汤组在骨髓4-HNE累积上显著减少。与对照组相比，AA组脾脏中铁死亡相关蛋白Acsl4表达升高，p-Stat3、p-Jak2表达上调；AA+鹿龙再生汤组Acsl4表达下调，p-Stat3、p-Jak2表达被抑制（*P*<0.05）。对照组、AA组、AA+鹿龙再生汤组外周血血浆IL-6的表达量分别为（65.60±6.01）、（166.50±3.32）、（119.37±4.29）pg/ml（*P*<0.05），AA+鹿龙再生汤组较AA组IL-6水平明显降低。

**结论:**

鹿龙再生汤通过调控铁死亡相关的基因Jak2/Stat3/Acsl4途径对AA模型小鼠具有显著的治疗效果。作用机制涉及抑制Jak2/Stat3信号通路以及调控Acsl4的表达，从而改善AA模型小鼠的造血功能。

## Abstract (en)

**Objective:**

To investigate the effect of Lulongzaisheng decoction on ferroptosis in aplastic anemia（AA）model mice and its potential mechanism of action on AA.

**Methods:**

Using female BALB/c mice as controls（control group）, a mouse model of AA was established by intraperitoneal injection of interferon-γ（IFN-γ）combined with oral gavage of busulfan. The AA group was treated with continuous oral gavage of Lulongzaisheng decoction for 10 days. The effects on the Jak2/Stat3/Acsl4 signaling pathway were assessed. Bone marrow biopsy and histopathological examination were performed to observe the degree of AA in each group of mice. Flow cytometry was used to detect the production of lipid reactive oxygen species（ROS）in the bone marrow cells. Immunohistochemical staining was performed to observe the expression of 4-HNE in the sternum. Western blot analysis was conducted to determine the protein expression levels of Jak2, p-Jak2, Stat3, p-Stat3, and Acsl4. ELISA was used to measure the levels of interleukin-6（IL-6）in the peripheral blood plasma.

**Results:**

Compared with the control group, mice in the AA group exhibited a reduction in hematopoietic tissues within the bone marrow cavity and significant fatty infiltration. In contrast, mice in the AA+Lulongzaisheng decoction group showed partial recovery of the bone marrow and a decrease in fatty infiltration. The lipid ROS proportions in the control, AA, and AA+Lulongzaisheng decoction groups were（47.01±3.07）％,（53.81±1.99）％, and（49.50±3.98）％, respectively（*P*<0.05）, indicating that the AA + Lulongzaisheng decoction group had a slightly lower accumulation of lipid ROS than the AA group. The expression areas of 4-HNE in the bone marrow cavity for the control, AA, and AA+Lulongzaisheng decoction groups were（6.34±1.07）％,（35.26±3.68）％, and（16.97±1.30）％, respectively（*P*<0.05）, demonstrating a significant reduction in 4-HNE accumulation in the bone marrow in the AA + Lulongzaisheng decoction group compared with that in the AA group. Compared with the control group, the AA group exhibited upregulated expression of the ferroptosis-related protein Acsl4, along with increased expression of p-Stat3 and p-Jak2. In the AA+ Lulongzaisheng decoction group, the expression of Acsl4 was downregulated, whereas those of p-Stat3 and p-Jak2 were inhibited（*P*<0.05）. The levels of IL-6 in the peripheral blood plasma were（65.60±6.01）,（166.50±3.32）, and（119.37±4.29）pg/ml in the control, AA, and AA+Lulongzaisheng decoction groups, respectively（*P*<0.05）. Compared with the AA group, the AA + Lulongzaisheng decoction group exhibited a significant decrease in IL-6 levels.

**Conclusion:**

This study shows that Lulongzaisheng decoction has a significant therapeutic effect on AA model mice by regulating the Jak2/Stat3/Acsl4 pathway. The mechanism of action of this traditional Chinese medicine involves inhibiting the Jak2/Stat3 signaling pathway and regulating the expression of Acsl4, thereby improving hematopoietic function in AA model mice. These findings provide new drug targets and treatment strategies for the treatment of AA.

再生障碍性贫血（AA）的病理特点是骨髓内造血细胞被脂肪细胞替代，脂肪细胞对造血干细胞的成熟与分化起到负性调控作用[1]。针对重型AA（SAA）患者，HLA相合同胞供者的造血干细胞移植（MSD-HSCT）仍然是40岁以下患者的首选治疗方案；对于不适合一线MSD-HSCT的患者，通常采用免疫抑制治疗（IST）联合TPO受体激动剂（TPO-RA），并可考虑增加其他促造血治疗[2]。鹿龙再生汤是朱良春先生针对AA的经典经验方，在临床上应用多年并取得良好效果[3]。我们的先前实验结果显示，鹿龙再生汤可以调节AA小鼠外周血单个核细胞及脾效应T细胞的T-bet和GATA-3基因表达[4]；能抑制AA小鼠CD4^+^CD25^+^调节性T细胞（Treg）Stat3的磷酸化并上调Foxp3表达，发挥免疫调节作用[5]；改善骨髓中红系爆式集落形成单位（BFU-E）和粒-单核系集落形成单位（CFU-GM）的生成[6]，最终促进血细胞的恢复。这些发现初步揭示了鹿龙再生汤在AA治疗中的作用机制。

铁死亡（Ferroptosis）是一种新型细胞程序性死亡形式，其特征是脂质活性氧（ROS）的致死性积聚，在形态学、生化和遗传学方面不同于其他传统形式的细胞死亡如凋亡、坏死和自噬[7]。近年来发现铁死亡在骨髓衰竭性疾病发病中起一定作用。本研究旨在观察鹿龙再生汤对AA小鼠铁死亡相关基因的影响，以进一步探讨该方改善AA小鼠造血功能的机制。

## 对象与方法

1. 实验研究对象：近交系雌性BALB/c小鼠，清洁型，8～12周龄，体重18～22 g，由南通大学医学院动物实验中心提供，合格证号：SCXK（苏）2020-0009。通过IFN-γ腹腔注射及白消安灌胃8 d诱导建立AA小鼠模型[8]。

2. 实验分组：取12只AA模型小鼠，随机分为2组，每组6只，造模后第8天开始灌胃处理，分别予PBS、鹿龙再生汤连续灌胃10 d，即为AA组、AA+鹿龙再生汤组。另取6只正常BALB/c雌性小鼠设为对照组，PBS连续灌胃10 d。每组灌胃量均为0.01 ml/g。

3. 实验仪器和试剂：RPMI 1640培养基、PBS（美国Gibco公司）；小鼠源性重组IFN-γ（美国Peprotech公司）；白消安、4-HNE抗体、H₂DCFDA染料（美国MCE公司）；红细胞裂解液、4％多聚甲醛（北京兰杰柯科技有限公司）；ELISA试剂盒（江苏艾迪生生物科技有限公司）；HE染料套装、EDTA抗原修复液（武汉赛维尔生物科技有限公司）；鹿龙再生汤（南通大学附属医院中医科）；兔抗小鼠GAPDH（杭州华安生物技术有限公司）；Jak2、p-Jak2、Stat3、p-Stat3一抗（美国CST公司）；Acsl4、Acsl3一抗（武汉三鹰生物技术有限公司）；Slc7a11一抗（上海萨博生物技术有限公司）；流式细胞仪（美国贝克曼库尔特公司）；Western blot仪（美国Bio-Rad公司）；冰冻切片机［赛默飞世尔科技（中国）有限公司］；酶标仪（美国BioTek公司）。

4. 骨髓组织病理形态学及免疫组化检测：骨髓组织标本常规方法进行固定、脱钙、脱水、石蜡包埋等操作，切成4～6 µm厚的切片。组织病理形态：HE染色，显微镜观察。免疫组化：将切片浸入pH 9.0的EDTA缓冲液中，微波高火煮沸后转低火维持10 min，经封闭及一抗、二抗孵育后，滴加DAB显色，显微镜观察后终止染色，PBS洗3次。苏木素复染细胞核3 min，蒸馏水冲洗，分化液分化5 s，自来水冲洗至返蓝。脱水、透明、封片，显微镜观察。

5. 流式细胞术检测脂质ROS：将一侧股骨置于RPMI 1640培养基的培养皿中，切除两端后，用1 ml注射器抽取RPMI 1640培养基冲洗骨髓，重复3～4次。冲洗液经尼龙滤网过滤，裂红后用PBS重悬细胞，再通过70 µm Falcon细胞滤网过滤，得到骨髓单个核细胞悬液。取500 µl悬液，加入0.5 µl H_2_ DCFDA染料，振荡混匀，室温避光孵育30 min。孵育后加入1 ml流式染色缓冲液，离心弃上清，用300 µl缓冲液重悬细胞后避光保存，上机检测。

6. Western blot：取0.1 g鼠脾脏组织于1.5 ml EP管，加1 ml裂解液（RIPA∶PMSF＝100∶1），冰上剪碎后超声破碎，冰上裂解30 min后离心取上清。用BCA法测蛋白浓度，根据蛋白浓度调整上样量后进行SDS-PAGE电泳，转膜后常温5％牛奶封闭2 h，PBS洗涤3次，每次15 min。加一抗4°C冰箱孵育过夜。次日PBS洗涤后室温孵育二抗2 h，再次洗涤，显影。实验重复3次。

7. ELISA：将小鼠血样从EDTA抗凝管转移至2 ml的EP管中，离心收集上层血浆。将铝箔袋在室温下平衡20 min后，取出所需酶标板条，设置标准品孔和样本孔，标准品孔各加不同浓度的标准品50 µl。样本孔先加待测样本10 µl，再加样本稀释液40 µl，空白孔不加。除空白孔外，标准品孔和样本孔中每孔加入辣根过氧化物酶（HRP）标记的IL-6检测抗体100 µl，用封板膜封住反应孔，37 °C恒温箱温育60 min。弃液体，吸水纸上拍干，每孔加满洗涤液，静置1 min，甩去洗涤液，吸水纸上拍干，重复洗板5次，接着每孔加入TMB底物A、B各50 µl，37 °C避光孵育15 min后加入终止液50 µl，使用酶标仪在450 nm波长处测定各孔的吸光度值，根据标准品的吸光度值绘制标准曲线，计算样品中IL-6的浓度。

8. 统计学处理：使用GraphPad Prism 10.2.3绘图并进行统计学。数据来自至少3次独立实验，结果以*x*±*s*表示，两组间比较采用双尾非配对*t*检验，多组间比较采用单因素方差分析，*P*<0.05为差异具有统计学意义。

## 结果

1. 小鼠胸骨组织病理学观察：骨髓HE染色示AA组小鼠骨髓损伤明显，髓腔内造血组织减少，出现较大脂滴空泡，脂肪化明显；AA+鹿龙再生汤组小鼠的骨髓有部分恢复，髓腔内造血组织有增加，脂滴空泡减少（图1）。

**图1:**
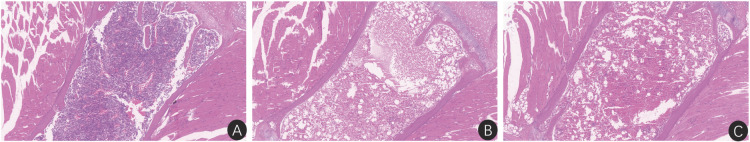
对照组、再生障碍性贫血（AA）组、AA+鹿龙再生汤组小鼠胸骨组织病理学结果（HE染色，低倍） **A** 正常BALB/c小鼠；**B** AA组小鼠；**C** AA+鹿龙再生汤组小鼠

2. 小鼠骨髓细胞脂质ROS水平的变化观察：流式细胞术检测结果提示AA组与对照组相比脂质ROS发生累积，AA+鹿龙再生汤组的脂质ROS累积量较AA组略低。对照组、AA组、AA+鹿龙再生汤组脂质ROS比例分别为（47.01±3.07）％、（53.81±1.99）％、（49.50±3.98）％（*P*<0.05）。

3. 小鼠胸骨免疫组织化学染色观察：与对照组相比，AA组小鼠胸骨中4-HNE表达显著升高，提示造模后氧化应激反应增强；AA+鹿龙再生汤组4-HNE表达水平降低，表明氧化应激反应得到部分缓解（图2）。对照组、AA组、AA+鹿龙再生汤组骨髓腔内4-HNE的表达面积分别为（6.34±1.07）％、（35.26±3.68）％、（16.97±1.30）％（*P*<0.05）。

**图2:**
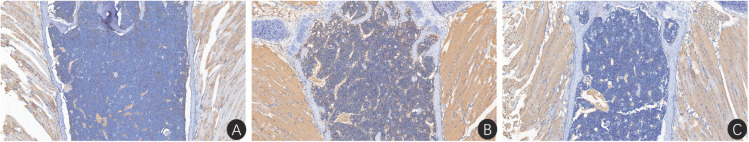
对照组、再生障碍性贫血（AA）组、AA+鹿龙再生汤组小鼠胸骨4-HNE免疫组化结果 **A** 正常BALB/c小鼠；**B** AA小鼠；**C** AA+鹿龙再生汤小鼠

4. 小鼠脾脏中铁死亡相关基因和Jak2/Stat3信号通路的蛋白表达比较：对照组、AA组、AA+鹿龙再生汤组Acsl3的相对表达水平分别为0.8±0.06、0.37±0.11、0.85±0.11；Acsl4的相对表达水平分别为0.96±0.13、1.24±0.12、0.81±0.15；Slc7a11的相对表达水平分别为1.02±0.05、0.54±0.14、0.95±0.04；Jak2的相对表达水平分别为0.18±0.03、0.44±0.02、0.44±0.01；p-Jak2的相对表达水平分别为0.51±0.05、1.35±0.11、0.62±0.06；Stat3的相对表达水平分别为0.77±0.07、1.27±0.08、0.66±0.07；p-Stat3的相对表达水平分别为0.38±0.04、1.17±0.17、0.46±0.04（图3）。与对照组相比，AA组铁死亡相关蛋白Acsl4表达升高，Acsl3、Slc7a11表达下降，p-Stat3、p-Jak2表达上调；AA+鹿龙再生汤组Acsl4表达下调，Acsl3、Slc7a11表达上调，p-Stat3、p-Jak2表达被抑制（*P*<0.05）。

**图3:**
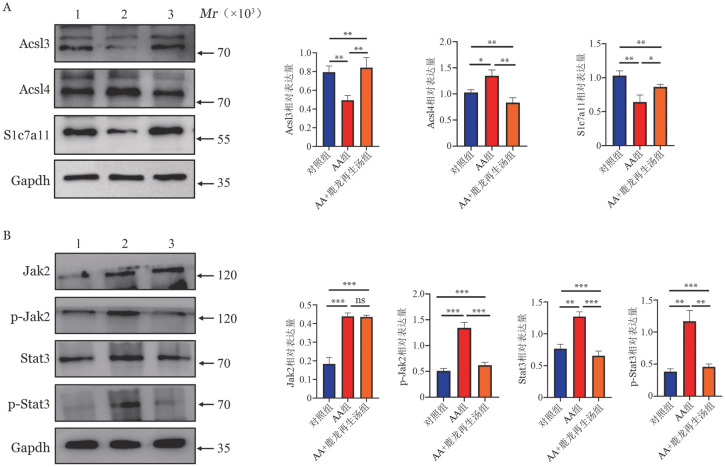
对照组、再生障碍性贫血（AA）组、AA+鹿龙再生汤组小鼠脾脏中铁死亡相关基因和Jak2/Stat3信号通路的蛋白表达水平（^ns^*P*≥0.05、*P<0.05、**P<0.01、***P<0.001） **注** １：对照组；２：AA组；3：AA+鹿龙再生汤组

5. 小鼠外周血血浆中IL-6的变化比较：对照组、AA组、AA+鹿龙再生汤组外周血浆IL-6的表达量分别为（65.60±6.01）、（166.50±3.32）、（119.37±4.29）pg/ml（*P*<0.05）。与对照组相比，AA组IL-6水平升高，而AA+鹿龙再生汤组IL-6水平降低。

## 讨论

AA是一种严重的骨髓衰竭性疾病，主要表现为骨髓造血功能受损、全血细胞减少，其发病机制涉及复杂的免疫反应，异常活化的T淋巴细胞在该过程中发挥着主导作用。近来的研究发现巨噬细胞在AA的发病过程中也起着重要作用[9]–[10]。我们过去研究通过AA模型小鼠实验观察到，巨噬细胞和病理性T细胞在疾病末期会归巢至骨髓，并表现出强烈的免疫攻击活性，伴随免疫抑制功能的显著下降[10]–[11]。AA患者在初诊时通常伴有血清铁和铁蛋白水平的显著升高，这一现象不仅与铁利用障碍有关，也与巨噬细胞在铁代谢中的作用相关。巨噬细胞通过回收衰老红细胞中的铁，导致Fe^2+^的过量积累，铁过载进一步抑制骨髓造血功能及加剧骨髓造血干细胞的铁死亡[12]–[13]。同时巨噬细胞与Stat3之间也存在密切联系。Stat3是一种关键的转录因子，广泛参与细胞增殖、分化、凋亡及免疫反应等多种生物过程，对细胞存活和生长具有促进作用。研究表明Stat3既能促进巨噬细胞的抗炎和免疫抑制功能，也能够调节其参与免疫反应的能力[14]。Stat3在铁死亡的发生中也起着重要作用，与多种与铁死亡相关的基因（如Slc7a11、Fth1、Acsl4、Gpx4）存在紧密的相互作用[15]–[17]。其中，Stat3的过度激活可能通过促进Acsl4的表达，进而促进脂质过氧化过程，推动铁死亡的发生[17]。Stat3还能调节铁代谢，尤其是在与铁调节蛋白的相互作用过程中，其激活可能通过改变铁的平衡，间接影响铁死亡的发生[18]。因Stat3的信号异常在AA的发病机制中起重要作用，而我们过去的研究已证实鹿龙再生汤能够通过抑制CD4^+^CD25^+^ Treg细胞中Stat3的磷酸化来调节免疫反应，进而改善造血功能，但其中具体下游作用机制仍不明确[5]。基于以上研究，本研究我们观察了鹿龙再生汤对Stat3下游及铁死亡通路的影响，旨在进一步探讨鹿龙再生汤改善造血的具体机制。

Jak-Stat通路在细胞信号转导中发挥着关键作用，尤其是在将外部化学信号传递至细胞核并调控DNA转录和活性方面。Jak2广泛表达于多种组织，特别是在骨髓、淋巴组织、肺和胆囊。Jak2通过结合同型二聚体受体调节骨髓细胞的增殖与存活，在造血干细胞的自我更新和分化中起重要作用[19]。Stat3的过度激活可能导致细胞异常增殖和凋亡调控失衡[20]。已有研究指出，Jak2/Stat3信号通路异常激活会影响红细胞的生成[21]，且与多种细胞因子（如IL-6和IL-11）的信号传递密切相关。在AA中，IL-6的水平通常会升高，这与炎症反应、免疫激活及骨髓抑制有关，IL-6不仅促进其他炎症细胞因子的释放，还可能对造血干细胞的功能和生存产生负面影响，从而干扰造血[22]。在AA患者中，Th1和Th17细胞群体通常表现出扩增和激活，而Th2和Treg细胞的数量和功能则下降。已有研究表明，抑制Jak2/Stat3通路能够调节炎症条件下Treg细胞的稳定性，并减少Th17细胞的数量和功能[23]，这与我们之前的研究鹿龙再生汤可恢复Treg细胞的功能的结论相一致。既往我们的研究还发现，AA模型中Th17的极化现象明显[24]，而最新的研究则揭示，极化的Th17细胞通过促炎细胞因子的分泌，能够进一步激活IL-6/Jak3/Stat3信号通路[25]。本实验我们观察到AA模型中IL-6水平升高、Jak2和Stat3的磷酸化被激活，推测可能是由于造血微环境中负性因子的增多，激活了Jak2/Stat3信号通路，从而抑制了骨髓造血功能。鹿龙再生汤可通过抑制IL-6的产生，进而抑制Jak2/Stat3信号通路的激活，恢复了骨髓造血功能。

铁死亡中脂质过氧化的累积是核心机制。PE作为一种关键磷脂，主要由花生四烯酸及其衍生物与乙醇胺组成，能够诱导细胞发生铁死亡。Acsl4在PE的合成和重塑中发挥着重要作用，其可激活多不饱和脂肪酸（PUFA），进而影响细胞膜的特性。当细胞内Acsl4的表达水平降低时，多不饱和脂肪酸的可用性减少，进而抑制脂质过氧化和铁死亡的发生[26]。Poindessous等[17]报道，Acsl4的启动子区域富含Stat3结合位点，Stat3可以调控Acsl4的表达。本实验我们观察到AA模型小鼠特异性铁死亡相关基因Acsl4上升，同时Stat3也出现了异常激活。因而我们推测AA小鼠异常激活的Stat3可能通过促进Acsl4的高表达，导致了造血细胞铁死亡的发生。在AA+鹿龙再生汤组中，Stat3的磷酸化水平降低，进一步影响了下游Acsl4的表达。因此推测鹿龙再生汤治疗AA的机制可能是通过抑制Stat3的磷酸化，间接减少Acsl4的表达，从而抑制铁死亡的发生而发挥作用的。

铁死亡过程中，脂质ROS的水平通常会升高。4-HNE作为重要脂质过氧化标志物也会大量积聚。其他相关基因和蛋白表达也会出现变化，如Acsl3、Slc7a11。本实验中我们还观察到AA小鼠模型4-HNE水平增加、脂质ROS水平的升高及特异性铁死亡标志物Acsl3和Slc7a11的表达水平下降，进一步确认了AA模型小鼠造血细胞铁死亡的存在。在AA+鹿龙再生汤组中，4-HNE水平下降，脂质ROS水平随之下降，其他特异性铁死亡标志物Acsl3和Slc7a11的表达水平上升，这也进一步证实了鹿龙再生汤可以通过减轻铁死亡，从而改善造血功能。

尽管本研究采用的是动物模型，但这些发现初步表明铁死亡可能是AA发病机制中的一个重要环节，而鹿龙再生汤可以通过减轻造血干细胞铁死亡改善造血，部分阐明了鹿龙再生汤治疗AA的机制。
